# Depth-dependent hysteresis in adhesive elastic contacts at large surface roughness

**DOI:** 10.1038/s41598-018-38212-z

**Published:** 2019-02-07

**Authors:** Weilin Deng, Haneesh Kesari

**Affiliations:** Brown University, School of Engineering, Providence, RI 02912 USA

## Abstract

Contact force–indentation depth measurements in contact experiments involving compliant materials, such as polymers and gels, show a hysteresis loop whose size depends on the maximum indentation depth. This depth-dependent hysteresis (DDH) is not explained by classical contact mechanics theories and was believed to be due to effects such as material viscoelasticity, plasticity, surface polymer interdigitation, and moisture. It has been observed that the DDH energy loss initially increases and then decreases with roughness. A mechanics model based on the occurrence of adhesion and roughness related small-scale instabilities was presented by one of the authors for explaining DDH. However, that model only applies in the regime of infinitesimally small surface roughness, and consequently it does not capture the decrease in energy loss with surface roughness at the large roughness regime. We present a new mechanics model that applies in the regime of large surface roughness based on the Maugis–Dugdale theory of adhesive elastic contacts and Nayak’s theory of rough surfaces. The model captures the trend of decreasing energy loss with increasing roughness. It also captures the experimentally observed dependencies of energy loss on the maximum indentation depth, and material and surface properties.

## Introduction

**Figure 1:**
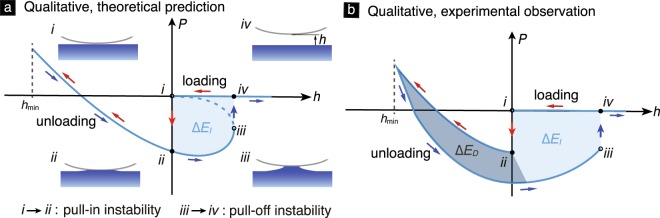
(**a**) The schematic of the *P*–*h* curve as per the JKR theory. The “pull-in” (*i* → *ii*) and “pull-off” (*iii* → *iv*) instabilities are marked along with the corresponding contact configurations. Closed and open symbols (circles) mark stable and unstable states on the *P*–*h* curve, respectively. A contact cycle includes the loading (red arrows) and unloading (blue arrows) phases. The size of the hysteresis loop formed in a contact cycle due to the instabilities (*i.e*., the shaded area Δ*E*_*I*_) denotes the hysteretic energy loss, which is depth independent. (**b**) The schematic of the *P*–*h* curve observed in some experiments [*e.g*., see Fig. 2(a)] which shows that the contact forces differ between the loading and unloading phases. The total hysteretic energy loss includes depth-independent part Δ*E*_*I*_ and depth-dependent part Δ*E*_*D*_.

**Figure 2:**
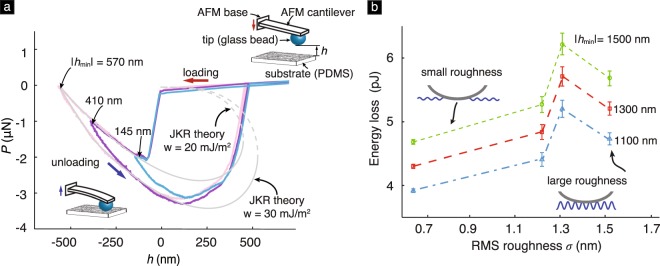
(**a**) Representative *P*–*h* curves measured in AFM contact experiments between a glass bead and a PDMS substrate^6^. The glass bead was of diameter ≈50 *μ*m. The PDMS sample was cast on a silicon wafer having a RMS roughness ≈1.3 nm. As can be noted, the measured *P*–*h* curves for the loading and unloading phases of the experiment are different. The size of the hysteresis loop increases with the maximum indentation depth, |*h*_min_|. The gray dashed curves are the fit of the loading and unloading branches of the measured *P*–*h* data to the JKR theory. (**b**) A plot showing the variation of total energy loss as function of the RMS roughness in the experiments. The RMS roughness refers to the surface roughness of the silicon wafer on which the PDMS substrates were cast. The indenting rate in the experiments corresponding to all data points shown in the plot was 1000 nm/s. See ref.^6^ for experimental details.

A clear understanding of adhesive contact mechanics is critical for spatially mapping out a material’s mechanical properties using, for example, nanoindentation- and contact mode atomic force microscopy (AFM)-based techniques^1,2^. Typically, material properties are measured by fitting contact force vs. indentation depth (*P*–*h*) measurements to a contact mechanics theory. Some of the most popular theories for modeling adhesive elastic contact include the Johnson–Kendall–Roberts (JKR)^3^, the Derjaguin–Muller–Toporov (DMT)^4^, and the Maugis–Dugdale (MD)^5^ theories. These classical contact theories predict that when the solids are in physical contact, the force is uniquely determined by the indentation depth and is independent of the history of the contact process [see Fig. 1(a)]. However, in many experiments it is found that the contact forces depend on the contact process history. A typical contact experiment consists of one or more contact cycles, each of which consisting of a loading and an unloading phase. In those phases the solids are, respectively, being moved towards and away from each other [Figs 1(a,b) and 2(a)]. It is found that, at a given indentation depth, the contact force differs depending on whether the experiment is in a loading or an unloading phase [see Figs 1(b) and 2(a)]. For example, Kesari *et al*.^6^ reported AFM-based contact experiments between a glass bead and a poly(dimethylsiloxane) (PDMS) substrate, which shows that the contact forces differ between the loading and unloading phases [Fig. 2(a)]. The force during the unloading phase was also observed to depend on the maximum indentation depth |*h*_min_| [Fig. 2(a)]. Kesari *et al*. termed this phenomenon depth-dependent hysteresis (DDH). The maximum indentation depth in a contact experiment is the indentation depth at the beginning of its unloading phase.

Depth-dependent hysteresis has also been observed in a number of other contact experiments, which span various length scales from *μ*m to cm and involve different soft materials such as gelatin, PDMS, and poly(n-butyl acrylate) (PNBA)^7–9^. When the solids are in contact, the classical contact theories predict a single *P*–*h* curve [Fig. 1(a)], whereas in the presence of DDH the experimental measurements display a different *P*–*h* branch for the loading and unloading phases [Figs 1(b) and 2(a)], respectively. The estimates for the material properties are different depending on which branch is chosen to be fitted to a classical contact theory. For example, fitting the unloading and loading branches of the *P*–*h* curves shown in Fig. 2(a) to the JKR theory yields values of 20 and 30 mJ/m^2^, respectively, for the Dupré’s work of adhesion *w*. Here *w* = *γ*_1_ + *γ*_2_ − *γ*_12_, where *γ*_1_ and *γ*_2_ are the surface energies of the two solids, respectively, and *γ*_12_ is the interfacial energy^10^. In some experiments, the ambiguity in the estimated values for *w* can be quite dramatic. For example, the *P*–*h* measurements reported by Guduru *et al*.^11^ for contact between a polycarbonate punch and a gelatin slab display significant DDH, with the measurements falling into distinct loading and unloading branches. Fitting the loading branch of those measurements to the JKR theory yields a value of 8 mJ/m^2^ for *w*, whereas fitting the unloading branch yields a value of 220 mJ/m^2^.

Depth-dependent hysteresis has been attributed to various mechanisms, such as the meniscus effect of ambient moisture^9^, the entanglement and interdigitation of tethered chains^12^, the formation of hydrogen bonds^13^, and the inelastic behaviors of materials (viscoelasticity^14^ and plasticity^15^). However, Kesari *et al*.^6^ showed that DDH persists even when the aforementioned mechanisms can be reasonably excluded. Motivated by the observation of overlapping hysteresis loops during consecutive load-unload cycles both in air and underwater, they hypothesized that DDH was due to the occurrence of a series of small-scale surface, mechanical instabilities that are created due to surface roughness, adhesion, and the large compliance of the soft materials involved^16^. Our recent static molecular simulations showed that this mechanism can operate in adhesive elastic contacts^17^. The surface instabilities through which small-scale roughness gives rise to DDH in the work of Kesari *et al*.^6^ and Kesari and Lew^16^ are the same as those through which surface undulations cause adhesive toughening in the work of Li and Kim^18^ and Guduru^19^.

The area enclosed by the *P*–*h* curves in a contact cycle, Δ*E*, is a measure of the energy lost during that cycle. It was found experimentally that Δ*E* initially increases and then later decreases with the surface roughness^6,20^, *e.g*., see Fig. 2(b). Kesari *et al*.^6,16^ presented a model for DDH that captures many of the salient features of DDH, including the initial increase of Δ*E* with the root mean square (RMS) roughness *σ*. However, that model does not capture the later decreases of Δ*E* with *σ*. Kesari *et al*. and we believe that this fact is due to the model’s assumption that the contact region is simply connected [top inset in Fig. 2(b)]. The contact region between two flat, perfectly smooth surfaces would be simply connected. It is likely to remain so even if infinitesimally small undulations were superimposed onto the flat surfaces. This would be especially true if the solids were composed of compliant materials, such as hydrogels or nonmineralized, biological tissues. However, irrespective of the compliance of the materials, as the height of the undulations is increased and the surface becomes rougher, the contact region will eventually become multiply connected [bottom inset in Fig. 2(b)].

**Figure 3:**
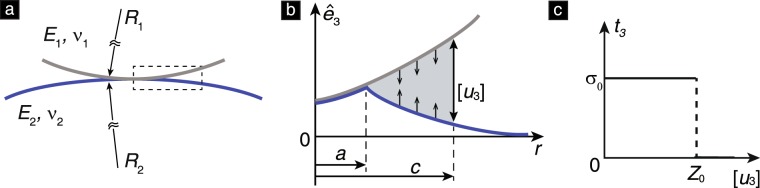
The MD model of adhesive elastic contact. (**a**) Geometry of the contacting solids. (**b**) The Dugdale cohesive zone, which is assumed to be present at the contact periphery (marked by the dashed box in (**a**)) as per the MD theory. The vector ${\hat{{\bf{e}}}}_{3}$ belongs to the set of Cartesian unit basis vectors, ${\{{\hat{{\bf{e}}}}_{i}\}}_{i=1,2,3}$, which is defined in Fig. 6(a). The symbol *r* denotes the radial coordinate in the plane spanned by ${\hat{{\bf{e}}}}_{1}$, ${\hat{{\bf{e}}}}_{2}$. The datum of *r* lies on the axial symmetry axis of the contacting solids. (**c**) A schematic diagram showing the traction distribution *t*_3_ as a function of the separation [*u*_3_]. The traction ${t}_{3}={\hat{{\bf{e}}}}_{3}\cdot ({\boldsymbol{\sigma }}{\hat{{\bf{e}}}}_{3})$, where ***σ*** is the Cauchy stress tensor. The parameters *Z*_0_ and *σ*_0_ are defined in the text.

**Figure 4:**
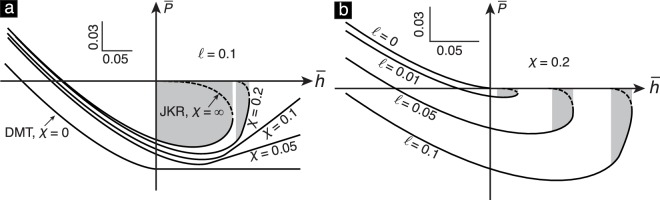
(**a**) The equilibrium *P*–*h* curves predicted by the MD theory for different *χ* values, with $\ell$ being held fixed at 0.1. The JKR and DMT limits are achieved when *χ* → ∞ and *χ* → 0, respectively. (**b**) The curves for different $\ell$ values, with *χ* being held fixed at 0.2. The Hertz limit is achieved when $\ell \to 0$. In both plots, the solid and dashed segments denote stable and unstable equilibrium states, respectively. The shaded area indicates the energy loss of the hysteresis loop.

**Figure 5:**
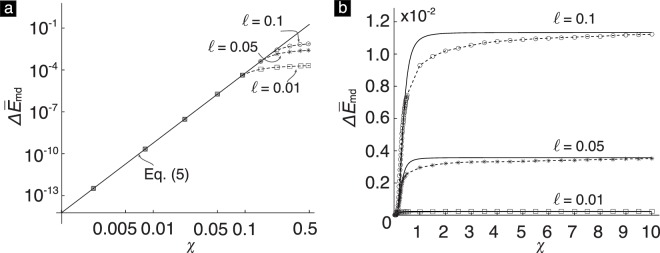
(**a**) The plot of the eq. (7) and the numerically computed ${\rm{\Delta }}{\bar{E}}_{{\rm{md}}}$ for different $\ell$ when *χ* is very small. (**b**) The comparison of exact values (dashed line with symbols) with approximate (solid line) values of ${\rm{\Delta }}{\bar{E}}_{{\rm{md}}}$ for different *χ* and $\ell$. The exact values are computed numerically using eqs (3)–(4b). The approximate values are computed from eq. (6).

In this work, we focus on the regime of large surface roughness where the contact region is multiply connected, and present a new model that captures the trend of Δ*E* decreasing with *σ*. This model is based on the MD theory of adhesive elastic contacts (Figs 3–5) and Nayak’s theory of rough surfaces^21^. The mechanism of energy loss in this model is similar to the one in the model presented by Kesari and Lew^16^, in which the energy loss arises as a consequence of small-scale surface mechanical instabilities. The primary difference between the model presented in^16^ and the new model herein is that the contact region in the former is simply connected whereas in the latter it is multiply connected.

**Figure 6:**
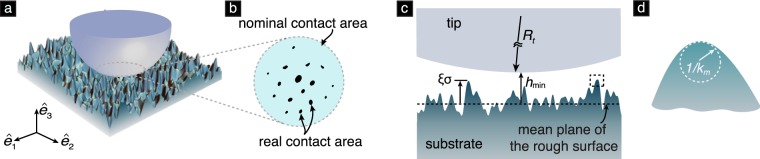
(**a**) The schematic of contact between a smooth rigid tip and a rough elastic substrate. (**b**) The schematic of nominal and real contact areas. (**c**) The section-view of the rough contact model shown in (**a**). (**d**) An asperity with radius of curvature 1/*k*_*m*_ at its apex as indicated by the dashed box in (**c**).

Our new model involves adhesive elastic contact between a smooth, rigid paraboloid (tip) and a rough, semi-infinite, deformable solid (substrate) [see Fig. 6(a)]. The substrate’s surface facing the tip is nominally flat but contains a random distribution of asperities. There are two major types of models used for studying contact between rough surfaces. The first type is based on the *non-interacting asperity contact model* pioneered by Greenwood and Williamson^22^, which is widely used for studying the effect of roughness on adhesion^23,24^, particle adhesion^25^, elasto-plastic contact^26^, and friction^27^. The second type is related to the *self-affine fractal contact model* put forward by Persson^28^. Ours is a non-interacting asperity type contact model, in which we assume that each substrate asperity interacts with the tip as though it were the only one interacting with it.

The energy loss Δ*E* was found experimentally to scale affinely with |*h*_min_|, with its minimum value corresponding to the case |*h*_min_| = 0. Furthermore, it was found that Δ*E* can be partitioned into two parts: a fixed part Δ*E*_*I*_ that only depends on the geometry and mechanical properties of the contacting solids and not on |*h*_min_|, and a variable part Δ*E*_*D*_ that in addition to the solids’ geometric and mechanical properties also depends on |*h*_min_| [see Fig. 1(b)]. The depth-independent part Δ*E*_*I*_ is a consequence of two surface mechanical instabilities that occur at the large-scale. These large-scale instabilities correspond to the initial sudden drop in the contact force [*i.e*., the transition from state (*i*) to (*ii*) in Fig. 1(b)] and the final abrupt increase in contact force [*i.e*., the transition from state (*iii*) to (*iv*) in Fig. 1(b)]. These instabilities are generally referred to as “pull-in” and “pull-off” instabilities. It was observed in the experiments^6^ that each of these large-scale instabilities always occurred only once in a contact cycle. Therefore, Δ*E*_*I*_ is the fixed, minimum amount of energy that gets dissipated in every contact cycle. Consequently, Δ*E*_*I*_ can be computed as the total energy dissipated in the contact cycle with |*h*_min_| = 0.

After the occurrence of the large-scale “pull-in” instability, as the solids are moved towards one another, more and more surface asperities will come into contact. We assume that those surface asperities will come into contact through small-scale surface mechanical instabilities, as done in refs^6,16^. Consequently, we refer to the asperities that come into contact during the loading phase after the occurrence of the large-scale “pull-in” instability as the *depth-dependent asperities*. Classical contact theories, which ignore roughness, predict that the contact radius prior to the occurrence of the large-scale “pull-off” instability is smaller than the one after the occurrence of the large-scale “pull-in” instability. We assume that that prediction holds true even in the presence of roughness and therefore during the unloading phase there will be a point when the contact region has receded back–in a nominal (large-scale) sense–to the one formed just after the occurrence of the large-scale “pull-in” instability. This implies that all the depth-dependent asperities would go out of contact before the occurrence of the large-scale “pull-off” instability. We assume that the detachment of the depth-dependent asperities takes place through the occurrence of small-scale instabilities, too. Thus, the energy loss Δ*E*_*D*_ consists of the energy lost during the instabilities through which the depth-dependent asperities come into and go out of contact. Since the larger the |*h*_min_| the larger will be the number of depth-dependent asperities, the energy loss Δ*E*_*D*_ increases with |*h*_min_|.

This paper is organized as follows. First, we evaluate the energy loss corresponding to the pair of small-scale “pull-in” and “pull-off” instabilities by using the MD theory. Second, based on Nayak’s theory of rough surfaces, we estimate the number of depth-dependent asperities and the depth-dependent energy loss Δ*E*_*D*_ during a contact cycle. Furthermore, we discuss the comparisons of the theoretical prediction of Δ*E*_*D*_ based on our model with the experimental measurements. Finally, we conclude by discussing the limitations of our model.

## Theory

### Energy loss per asperity using the Maugis-Dugdale theory

The MD theory describes the axi-symmetric contact between two isotropic, homogeneous, linear elastic solids of Young’s moduli and Poisson’s ratios *E*_*i*_ and *ν*_*i*_ (*i* = 1, 2), respectively [see Fig. 3(a)]. The adhesive interactions are introduced using the *Dugdale cohesive zone* model^29^. As per this model, a surface material point experiences a traction only if its distance from the other solid in the direction normal to the surface is less than *Z*_0_. Thus *Z*_0_ denotes the range of the inter-body adhesive forces, which are thought to arise from van der Waals-type interactions between the surfaces. When the normal distance of the material point is less than *Z*_0_ but non-zero, then the traction it experiences is purely tensile and of a fixed magnitude of *σ*_0_ [see Fig. 3(b,c)].

The contact process is governed by the two dimensionless parameters

1 $$\ell =\frac{\pi w}{{E}^{\ast }R}$$

and

2 $$\chi =\frac{w}{{E}^{\ast }{Z}_{0}},$$

where 1/*R* = 1/*R*_1_ + 1/*R*_2_ is the sum of the mean curvatures of the contacting solids at their respective points of contact, and $\mathrm{1/}{E}^{\ast }=\mathrm{(1}-{\nu }_{1}^{2})/{E}_{1}+\mathrm{(1}-{\nu }_{2}^{2})/{E}_{2}$. In terms of these non-dimensional parameters, the magnitude of the contact force *P* and the indentation depth *h* at equilibrium are related as

3 $$\begin{array}{rcl}\bar{P} & = & \{\begin{array}{ll}\frac{2}{3}{\bar{a}}^{3}-\chi {\bar{a}}^{2}(\sqrt{{m}^{2}-1}+{m}^{2}\,{\tan }^{-1}\sqrt{{m}^{2}-1}), & \bar{a} > \mathrm{0,}\\ -\frac{\pi }{2}\chi {\bar{c}}^{2}, & \bar{a}=\mathrm{0,}\end{array}\\ \bar{h} & = & \{\begin{array}{ll}-{\bar{a}}^{2}+2\chi \bar{a}\sqrt{{m}^{2}-1}, & \bar{a} > \mathrm{0,}\\ 2\chi \bar{c}+{\bar{h}}_{g}, & \bar{a}=\mathrm{0,}\end{array}\\ \ell  & = & \{\begin{array}{ll}\begin{array}{c}{\bar{a}}^{2}[\sqrt{{m}^{2}-1}+({m}^{2}-\mathrm{2)}{\tan }^{-1}\sqrt{{m}^{2}-1}]\\ \,+4\bar{a}{\chi }^{2}[\sqrt{{m}^{2}-1}\,{\tan }^{-1}\sqrt{{m}^{2}-1}-m+1],\end{array} & \bar{a} > \mathrm{0,}\\ \frac{\pi }{2}\chi {\bar{c}}^{2}+\mathrm{2(}\pi -\mathrm{2)}{\chi }^{2}\bar{c}+\pi \chi {\bar{h}}_{g}, & \bar{a}=\mathrm{0,}\end{array}\end{array}$$

where $\bar{P}=P/(2{E}^{\ast }{R}^{2})$, $\bar{h}=h/R$, ${\bar{h}}_{g}={h}_{g}/R$, $\bar{a}=a/R$, $\bar{c}=c/R$ and *m* = *c*/*a*. The parameter *c* is defined such that all surface points whose radial coordinate in the undeformed configuration *r* is less than or equal to *c* experience a non-zero traction force [see Fig. 3(b)]. The coordinate system corresponding to *r* is defined in Fig. 3. The parameter *a* is defined such that there is no separation, [*u*_3_], between the solids’ surfaces in the region *r* ≤ *a*. The separation [*u*_3_] is defined in Fig. 3(b) and is usually referred to as the *crack opening displacement*^10^. The parameter *h*_*g*_ is the separation between the solids’ surface points at *r* = 0 when *a* = 0. Due to the finite range of the inter-body adhesive interactions, the surface tractions in the MD theory do not vanish when $\bar{a}\to 0$, which is the case in the JKR and Hertz theories. For this reason we refer to *c* as the contact radius. The cases $\bar{a} > 0$ and $\bar{a}=0$ in eq. (3) were, respectively, derived by Maugis^5^ and Kim *et al*.^30^.

Figure 4 shows the representative equilibrium *P*–*h* curves for different combinations of parameters *χ* and $\ell$ according to eq. (3). When $\ell  > 0$, the MD theory asymptotes to the JKR and DMT theories, respectively, as *χ* → ∞ and 0 [Fig. 4(a)]. The JKR theory applies to compliant materials having a large work of adhesion, while the DMT theory applies to stiff materials having a small work of adhesion. When *χ* is any finite, fixed value, then as $\ell \to 0$ the MD theory asymptotes to the Hertz theory [Fig. 4(b)].

When $\ell  > 0$, the MD theory predicts that the solids will come into and go out of contact through the well-known mechanical instabilities termed the “pull-in” and “pull-off” instabilities during a contact cycle. The schematic of a typical equilibrium *P*–*h* curve predicted by the MD theory is shown in Fig. 1(a). In that schematic, the “pull-in” and “pull-off” instabilities, respectively, correspond to the initial sudden drop in the contact force [state (*i*) to (*ii*)] and the final sudden increase in the contact force [state (*iii*) to (*iv*)]. In a displacement controlled experiment, the measured *P*–*h* curve will be the envelope of the equilibrium *P*–*h* curve. The energy lost during a contact cycle, Δ*E*_m*d*_, due to the “pull-in” and “pull-off” instabilities, is equal to the area enclosed by the *P*–*h* curves measured during that cycle. It is denoted as the shaded area in Fig. 4.

The energy loss can be computed from the $\bar{P}$–$\bar{h}$ curves, which are defined by eq. (3), as

4a $${\rm{\Delta }}{E}_{{\rm{m}}{\rm{d}}}=2{E}^{\ast }{R}^{3}{\rm{\Delta }}{\bar{E}}_{{\rm{m}}{\rm{d}}},$$

where

4b $${\rm{\Delta }}{\bar{E}}_{{\rm{md}}}={\int }_{{r}_{i}}^{{r}_{o}}\bar{P}(r)\frac{\partial \bar{h}(r)}{\partial r}\,dr.$$

The limits of integration *r*_*i*_ and *r*_*o*_ in eq. (4b) are the contact radii at the instances just after the occurrence of the “pull-in” instability and before the occurrence of the “pull-off” instability, respectively. We refer to ${\rm{\Delta }}{\bar{E}}_{{\rm{md}}}$ as the normalized energy loss. Since the $\bar{P}$–$\bar{h}$ curves are completely defined by *χ* and $\ell$, the energy loss ${\rm{\Delta }}{\bar{E}}_{{\rm{md}}}$ only depends on these two parameters, too. We could not find a closed form expression for ${\rm{\Delta }}{\bar{E}}_{{\rm{md}}}$ by evaluating the integral in eq. (4b) analytically for arbitrary values of *χ* and $\ell$. However, we were able to obtain closed form expressions in three special cases. When *χ* → ∞, with $\ell$ held fixed, we find that

5a $${\rm{\Delta }}{\bar{E}}_{{\rm{md}}} \sim 0.5262{\ell }^{\mathrm{5/3}}.$$

On the other hand, when χ → 0 with *ℓ* held fixed we obtain that

5b $${\rm{\Delta }}{\bar{E}}_{{\rm{md}}} \sim 5.8483{\chi }^{5},$$

as shown in Fig. 5(a). Finally, when $\ell \to 0$ with *χ* held fixed, the energy loss $\Delta {\bar{E}}_{{\rm{m}}d}\to 0$.

We numerically compute ${\rm{\Delta }}{\bar{E}}_{{\rm{md}}}$ for a wide range of *χ* and $\ell$ values (see Fig. 5). As can be seen, ${\rm{\Delta }}{\bar{E}}_{{\rm{md}}}$ increases with both *χ* and $\ell$. By analyzing the numerical data shown in Fig. 5, we find that the dependence of ${\rm{\Delta }}{\bar{E}}_{{\rm{md}}}$ on *χ* and $\ell$ can be well approximated by the values of the empirical function

6 $${\rm{\Delta }}{\tilde{E}}_{{\rm{md}}}(\chi ,\ell )=\frac{{c}_{1}{\chi }^{5}}{{[{c}_{2}({\chi }^{3}/\ell )+1]}^{\mathrm{5/3}}},$$

where *c*_1_ = 5.8483 and *c*_2_ = 4.2415. A comparison of the approximate values of ${\rm{\Delta }}{\bar{E}}_{{\rm{md}}}$ given by eq. (6) with its exact values computed numerically is shown in Fig. 5(b). A notable aspect of the empirical function ${\rm{\Delta }}{\bar{E}}_{{\rm{md}}}$ is that it gives the exact values of ${\rm{\Delta }}{\bar{E}}_{{\rm{md}}}$ in the limit *χ* → 0 and *χ* → ∞, while holding $\ell$ fixed, and also in the limit $\ell \to 0$, while holding *χ* fixed. The differences between ${\rm{\Delta }}{\bar{E}}_{{\rm{md}}}$ and ${\rm{\Delta }}{\tilde{E}}_{{\rm{md}}}$ are more noticeable at intermediate values of *χ*. However, we found those differences to be less than 15% for the data shown in Fig. 5. Therefore, we will approximate ${\rm{\Delta }}{\bar{E}}_{{\rm{md}}}$ with ${\rm{\Delta }}{\tilde{E}}_{{\rm{md}}}$ in our remaining analysis.

### Depth-dependent energy loss due to the asperity level instabilities

In this section we present a rough surface contact model, and use that model to compute the depth-dependent part of the energy loss, Δ*E*_*D*_, as the product of the total number of depth-dependent asperities and the mean energy loss per asperity.

In our rough surface contact model, the tip is a paraboloid with the radial profile ${\mathop{u}\limits^{ \sim }}_{3}=h+{r}^{2}/(2{R}_{t})$, *r* ∈ [0, *R*_*t*_]. We describe the geometry of our model using the Cartesian coordinates *x*_*i*_, *i* = 1, 2, 3, whose corresponding basis vectors, ${\hat{{\bf{e}}}}_{i}$, are shown in Fig. 6(a).We describe the substrate’s surface topography using the function $z\,:{{\mathbb{R}}}^{2}\to {\mathbb{R}}$, which gives the height (*x*_3_ coordinate) of the substrate’s surface points as a function of their *x*_1_, *x*_2_ coordinates. The datum of the ${\hat{{\bf{e}}}}_{3}$ direction is chosen such that ${\int }_{{{\mathbb{R}}}^{2}}z({x}_{1},{x}_{2})\,d{x}_{1}d{x}_{2}=0$. That is, the set of points *x*_3_ = 0 form the mean plane of the substrate’s rough surface [see Fig. 6(c)]. The datums of the ${\hat{{\bf{e}}}}_{1}$, ${\hat{{\bf{e}}}}_{2}$ directions are chosen such that the coordinate system’s origin is the point where the tip’s rotational symmetry axis intersects the mean plane.

Consider a region in the mean plane having an area of unit magnitude. We say that this unit region contains an asperity whose apex has the coordinates (*x*_1_, *x*_2_, *z* (*x*_1_, *x*_2_)), if it contains the point (*x*_1_, *x*_2_, 0). The unit region will, in general, contain a large number of asperities. A number of surface topography measurements have shown that the variation of a rough surface’s geometric features can be well described using stochastic models^31,32^. Motivated by those results, we model the variation of the different geometric characteristics of the asperities belonging to the unit region using the probability density functions (PDFs) given by Nayak^21^. In our current model, we assume that, in a statistical sense, the substrate’s surface roughness is homogeneous and isotropic. That is, the PDFs characterizing the different geometric features of the asperities do not depend on the location or the orientation of the unit region. For this special case, Nayak^21^ gives the joint PDF of the heights and curvatures of the asperities belonging to the unit region to be

7 $$p(\xi ,t)=\frac{\sqrt{3{C}_{1}}}{2\pi }{e}^{-{C}_{1}{\xi }^{2}}({t}^{2}-2+2{e}^{-\frac{{t}^{2}}{2}}){e}^{-\frac{{C}_{1}{t}^{2}+{C}_{2}t\xi }{2}},$$

where *ξ* ∈ (−∞, ∞) is the asperity height normalized by the surface’s RMS roughness *σ* [see Fig. 6(c)], $t=-\,\sqrt{\mathrm{3/}{m}_{4}}{k}_{m}$, and the asperity curvature *k*_*m*_ ∈ (0, ∞) is the surface’s mean curvature at the apex of an asperity [see Fig. 6(c,d)]. The constants *C*_1_, *C*_2_ in eq. (7) are defined as ${C}_{1}\,:\,=\alpha /(2\alpha -\mathrm{3)}$ and ${C}_{2}\,:\,={C}_{1}\sqrt{\mathrm{12/}\alpha }$, where *α* is an important parameter called Nayak’s parameter. It is defined as $\alpha \,:\,={m}_{0}{m}_{4}/{m}_{2}^{2}$, where *m*_0_, *m*_2_, and *m*_4_ are the surface’s spectral moments. These moments can be computed from the equation

8 $${m}_{n}=\frac{2\sqrt{\pi }{\rm{\Gamma }}\mathrm{((1}+n\mathrm{)/2)}}{{\rm{\Gamma }}\mathrm{(1}+n\mathrm{/2)}}{\int }_{0}^{\infty }\,{q}^{n+1}{C}^{{\rm{iso}}}(q)\,dq$$

by setting *n* = 0, 2 and 4, respectively, where Γ is the gamma function, the function ${C}^{{\rm{iso}}}:{\mathbb{R}}\to {\mathbb{R}}$; is the isotropic surface’s power spectral density (PSD). It is determined by the surface’s topography *z*. See Supplementary Material for its complete definition.

We assume in our model that the contact between the tip and the substrate takes place only at the asperities. Consequently, in our model the real contact region is smaller than the nominal contact region. We define the nominal contact region to be a circular region in the mean plane that contains all the contacting asperities [Fig. 6(b)]. The nominal contact region is also referred to as the apparent contact region in the literature, since at the large-scale it is the region over which the solids appear to be in contact. The nominal contact region grows and recedes during the loading and unloading phases of a contact cycle, respectively. The evolution of the real contact region is much more complicated. The definition of the nominal contact region, by itself, does not imply that all asperities contained within it are in contact with the tip. Indeed, it is possible that many asperities never make contact despite belonging to the nominal contact region during some instance of the contact cycle. However, as part of our model, we assume that all asperities within a nominal contact region make and break contact with the tip as that region forms and unforms. As a consequence of this assumption, the total number of depth-dependent asperities can be computed as the product of the asperity density and the area of the nominal contact region that forms after the occurrence of the large-scale “pull-in” instability during the remainder of the contact cycle’s loading phase. The asperity density is the total number of asperities contained in a nominal contact region of unit area. Nayak^21^ gives the total number of asperities contained in a region of the mean plane of unit area to be

9 $$\eta =\frac{{m}_{4}}{6\pi \sqrt{3}{m}_{2}}.$$

Recall that the nominal contact region is part of the mean plane. Therefore, *η* is in fact equal to the asperity density. We compute the area of the nominal contact region formed after the occurrence of the large scale “pull-in” instability as

10 $${\rm{\Delta }}{A}_{c}={A}_{c}^{{h}_{{\rm{\min }}}}-{A}_{c}^{0},$$

where ${A}_{c}^{0}$ and ${A}_{c}^{{h}_{{\rm{\min }}}}$ are areas of the nominal contact region at the large-scale “pull-in” instability point (*i.e*., *h* = 0, marked as state *ii* in Fig. 1(b)) and at the maximum indentation depth (*i.e*., *h* = *h*_min_, see Fig. 1(b)), respectively. Our rough contact model does not provide predictions for the nominal contact region. In many contact experiments the nominal contact region is measured as part of the experiment (*e.g*., see Guduru and Bull^11^). In such cases, the total number of depth-dependent asperities can be estimated by using the measured nominal contact area values in conjunction with eqs (9) and (10). In other situations, where such measurements are unavailable we believe that the best alternative is to estimate the nominal contact region using a classical adhesive elastic contact theory. For example, in the next section, we estimate the nominal contact region in the experiments of Kesari *et al*. using the JKR theory.

We estimate the energy loss for a single depth-dependent asperity, Δ*E*_md_, using the MD theory. That energy loss is not constant between the asperities, but varies between them depending on their curvature. Using eq. (7), we find that the variation of curvatures in the population of all asperities contained in any unit region of the mean plane to be

11 $${p}_{\kappa }(t)=\sqrt{\frac{3}{4\pi }}({t}^{2}-2+2{e}^{-\frac{{t}^{2}}{2}}){e}^{-\frac{(8{C}_{1}^{2}-{C}_{2}^{2}){t}^{2}}{16{C}_{1}}}.$$

Recall that the nominal contact region belongs to the mean plane. Therefore, the PDF eq. (11) also applies to the population of all the asperities contained in any nominal contact region of unit area. Since the depth-dependent asperities are the total number of asperities contained in the nominal contact region formed after the occurrence of the large scale “pull-in” instability, eq. (11) also applies to the population of all depth-dependent asperities. Thus, the mean energy loss per depth-dependent asperity can be computed as

12 $$\langle {\rm{\Delta }}{E}_{{\rm{md}}}\rangle ={\int }_{-\infty }^{0}\,{\rm{\Delta }}{E}_{{\rm{md}}}{p}_{\kappa }(t)\,dt.$$

Writing Δ*E*_md_ in eq. (12) in terms of ${\rm{\Delta }}{\bar{E}}_{{\rm{md}}}$ using eq. (4a), and then approximating ${\rm{\Delta }}{\bar{E}}_{{\rm{md}}}$ with ${\rm{\Delta }}{\bar{E}}_{{\rm{md}}}$ defined in eq. (6), we get

13 $$\langle {\rm{\Delta }}{E}_{{\rm{md}}}\rangle \approx 2{E}^{\ast }{\int }_{-\infty }^{0}\,{\rm{\Delta }}{\tilde{E}}_{{\rm{md}}}(\chi ,\ell (t)){p}_{\kappa }(t){R}^{3}(t)\,dt,$$

where

14a $$\ell (t)=\frac{\pi w}{{E}^{\ast }R(t)},$$

14b $$R(t)={(\frac{1}{{R}_{t}}-\sqrt{\frac{{m}_{4}}{3}}t)}^{-1}.$$

Equation (14a) follows from noting that in eq. (1) the second argument, $\ell$, of the function ${\rm{\Delta }}{\bar{E}}_{{\rm{md}}}$ depends on the effective mean curvature 1/*R*, which is the sum of mean curvatures of the solids at their respective points of contact. We assume that at all contact points the tip’s curvature equals 1/*R*_t_ and that the asperity’s curvature at the contact point is the same as its curvatures *k*_*m*_ at its apex. The eq. (14b) follows these assumptions and the fact that ${k}_{m}=-\,\sqrt{{m}_{4}/3}t$. Multiplying the mean energy loss per depth-dependent asperity with the total number of those asperities we get

15 $${\rm{\Delta }}{E}_{D}=\eta {\rm{\Delta }}{A}_{c}\langle {\rm{\Delta }}{E}_{{\rm{md}}}\rangle ,$$

where *η*, Δ*A*_*c*_, and 〈Δ*E*_md_〉 are, respectively, given by eqs (9), (10), and (13).

## Comparison with Experiments

**Figure 7:**
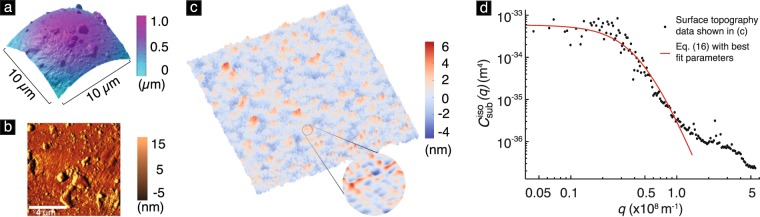
(**a**) The curved shape of the tip (which is a glass bead) and (**b**) its surface topography after subtracting the curvature effect. The RMS roughness of this bead is 12.14 nm. See Table 1 of Supplementary Material for details about the glass bead roughness. (**c**) The surface topography of the Si mold scanned over a area of 2 *μ*m × 2 *μ*m with a total of 256 points in each direction. The measured RMS roughness *σ* is 1.52 nm. (**d**) The power spectrum of the Si mold’s surface topography, and the corresponding fitting to the PSD function eq. (16) with best fitting parameters *σ* = 1.45 nm, *L* = 20.1 nm and *n* = 3.28.

In this section, we use eq. (15) to estimate Δ*E*_*D*_ in the glass bead–PDMS contact experiments reported by Kesari *et al*.^6^ and compare the estimates with measured values. The experiments involved contact between a spherical glass bead and PDMS substrates. The geometry of the contacting solids in the experiments is shown in the insets of Fig. 2(a). In the experiments, both the substrate and the tip are rough [see Fig. 7(a–c)]. However, in our model only the substrate is rough. This makes the quantitative comparison of our model with the experiments challenging. Nevertheless, we still attempt to compare our model’s predictions with the experiments by taking our model’s surface topography function to be a scalar multiple of that of the substrate so that the substrate’s roughness in our model stands in for the roughness of both the tip and the substrate in the experiments. Despite its crude nature, we hope that some knowledge can yet be gained about the utility of our model from this comparison.

To calculate Δ*E*_*D*_ from eq. (15), we need to know the asperity density *η*, the mean energy loss per asperity 〈Δ*E*_md_〉, and the nominal contact area Δ*A*_*c*_ in the context of the experiments. To calculate the first two of these quantities we need to know the *C*^iso^ function. Given a substrate surface topography function, we are able to numerically compute *C*^iso^(*q*), where *q* is the wavenumber magnitude, using the method presented in ref.^33^, see §1 of Supplementary Material for details. We take the surface topography function in our model to be a scaled version of the surface topography function of the substrate in the experiments.

Kesari *et al*. reported that it was difficult to measure the PDMS substrates’ surface topography directly because of their low stiffness. As an alternative, they assumed that a substrate’s topography function was the same as that of the Si mold on which it was cast. For the purposes of the current comparison, we make the same assumption. However, as we discuss in § Concluding remarks, the validity of this assumption needs further investigation.

The PDMS substrates were cast on four different Si molds having different surface topographies. The RMS roughness of those topographies ranged between 0.65 nm and 1.52 nm [see, *e.g*., Fig. 2(b)]. Since our model applies to the large surface roughness regime, we only consider the experiments on the substrate with the largest roughness, namely 1.52 nm. Figure 7(c) shows the surface topography of the Si mold corresponding to this substrate. We use that topography data to construct the surface topography function for the substrate in the experiments. The values of *C*^iso^ corresponding to this surface topography function are shown in Fig. 7(d). As can be seen, the values of *C*^iso^ are approximately constant at small wavenumber magnitudes, and fall off rapidly at large wavenumber magnitudes. This behavior is similar to that of a power-law PSD function. To be specific, consider the PSD function

16 $$q\mapsto \frac{1}{{C}_{0}}\frac{{e}^{-q/{q}_{0}}{L}^{2}{\sigma }^{2}}{{\mathrm{(1}+{L}^{2}{q}^{2})}^{n}},$$

where

$${C}_{0}=2\pi {\int }_{0}^{\infty }\,\frac{{e}^{-q/{q}_{0}}{L}^{2}q}{{\mathrm{(1}+{L}^{2}{q}^{2})}^{n}}\,dq,$$

and *L*, *q*_0_, and *n* are parameters referred to as the correlation length, cut-off wavenumber, and the power-law index, respectively. The PSD function (16) is a modified version of the *k*-correlation model, or *ABC* model, which has been shown to be applicable to a large variety of surface topographies^34,35^. We found that the PSD function (16) matches the *C*^iso^ function whose values are shown in Fig. 7(d) remarkably well for the parameter values

17 $$\sigma =1.45\,{\rm{nm}},\,L=20.1\,{\rm{nm}},\,n=\mathrm{3.28,}\,{\rm{and}}\,{q}_{0}=1.0\times {10}^{8}\,{{\rm{m}}}^{-1}.$$

(The values for *σ*, *L*, and *n* were obtained by minimizing a measure of the difference between the *C*^iso^ values shown in Fig. 7(d) and the values given by the PSD function eq. (16). The value for *q*_0_ was chosen independently before performing the minimization.) Therefore, we take the PSD function (16) with the values for the parameters in it given by eq. (17) to be an analytical representation of the *C*^iso^ function that corresponds to the surface topography of the substrate in the experiments.

To account for the roughness of the tip in the experiments in our model, in which the tip is always smooth, we take the surface topography function of the substrate in our model to be a scaled version of that of the substrate in the experiments. Specifically, if in the experiments the height of a substrate surface point is *z*(*x*_1_, *x*_2_) then in our model we take the height of that point to be *kz*(*x*_1_, *x*_2_), where *k *≥ 1. Taking *k* = 1 would amount to simply ignoring the roughness of the tip-in-the-experiments in our model. We perform our comparison for a range of *k* values, which–as we shall discuss shortly–we selected by taking into consideration the measured roughness of the tip-in-the-experiments. It follows from §1 of Supplementary Material that if the isotropic PSD function corresponding to *z* is *C*^iso^, then that corresponding to *kz* is *k*^2^ *C*^iso^. Therefore, we take the *C*^iso^ function in our model to be *k*^2^ times the PSD function (16) with the values for the parameters in it still being given by eq. (17).

Knowing the *C*^iso^ function in our model, we numerically evaluate the integrals in eq. (8) for the cases *n* = 0, 2 and 4 to get

18 $${m}_{0}\approx 2.1\,{k}^{2}\,{{\rm{nm}}}^{2},\,{m}_{2}\approx 1.3\,{k}^{2}\times {10}^{-3},\,{\rm{and}}\,{m}_{4}\approx 4.3\,{k}^{2}\,{\mu {\rm{m}}}^{-2}.$$

Substituting the values for the spectral moments given in (18) in eq. (9) we get the asperity density *η* ≈ 101.4 *μ*m^−2^.

The mean energy loss 〈Δ*E*_m*d*_〉 can be computed from eqs (13)–(14) on knowing the values of *R*, $\ell$, and *p*_*κ*_ for any given t < 0 and the parameter *χ*. We calculate the values of *R* from (14b), in which we take *R*_*t*_ to be 25 *μ*m, as that was the radius of the glass bead (tip) in the experiments, and *m*_4_’s value to be that which was given in eq. (18). Knowing *R*, we calculate the values of $\ell$ from eq. (14a). In eq. (14a), as well as in the remainder of this comparison, we take the material properties E^*^ and *w* to be, respectively, 0.75 MPa and 26 mJ/m^2^, since these were the values measured for them in the experiments. We calculate *α* from the values for the spectral moments given in eq. (18) and then use it to calculate the parameters *C*_1_ and *C*_2_. Knowing these parameters, we calculate the values of *p*_*κ*_ from eq. (11). Having knowledge of E^*^ and *w* the parameter *χ* can be computed from eq. (2) once we have knowledge of *Z*_0_. However, we could not find a clear way to identify *Z*_0_ in the experiments. Therefore, we treat *Z*_0_ as a fitting parameter in our comparison.

Unfortunately, neither Kesari *et al*. reported measurements of Δ*A*_*c*_ in their experiments nor does our model give predictions for it. For a lack of a better alternative, we use the JKR theory to estimate Δ*A*_*c*_ in the experiments. The JKR theory is the most widely used model for adhesive elastic contact, which only applies to contact between smooth surfaces. Kesari and Lew^16^ presented a generalization of the JKR theory that applies to contact between rough surfaces and gives a prediction for the nominal contact area. However, as we discussed in the introduction, that model only applies in the regime of small surface roughness. Employing the JKR theory we find that Δ*A*_*c*_ in the experiments is approximately equal to 4*R*_*t*_|*h*_min_|. See §2 of Supplementary Material for details.

**Figure 8:**
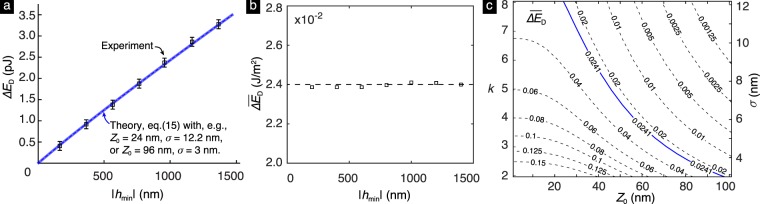
(**a**) The comparison of the depth-dependent energy loss Δ*E*_*D*_ measured in the experiment^6^ (squares) with the estimations based on the theoretical model according to eq. (15) with *Z*_0_ = 24 nm, *σ* = 12.2 nm (thick, solid line) and *Z*_0_ = 96 nm, *σ* = 3 nm (thin, dashed line). The error bars indicate the standard deviation of the measurements of hysteretic energy loss taken at five locations on the PDMS substrates in the experiments. (**b**) The plot of depth-dependent energy loss per unit nominal contact area ${\overline{{\rm{\Delta }}E}}_{D}$ with |*h*_min_|. It is essentially a constant equal to 0.0241 J/*m*^2^ (dashed line). (**c**) The contour plot of ${\overline{{\rm{\Delta }}E}}_{D}$ as a function of *Z*_0_ and *σ*. The contour of the experimental value of ${\overline{{\rm{\Delta }}E}}_{D}$ is shown as a solid blue line. Also, while making the comparison if we choose the value of *k* (equivalently *σ*) to be the ordinate of a point lying on the blue line then the best fit value for *Z*_0_ in the comparison is the abscissa of that point.

The experimentally measured Δ*E*_*D*_ values reported by Kesari *et al*. are shown in Fig. 8(a). As the final step for making the comparison, we need to choose appropriate values for *k*. Recall that we introduced the scaling parameter *k* to account for the roughness of the tip in the experiments in our model, in which the tip is smooth. The tip in the experiments was a spherical glass bead. Table 1 in Supplementary Material gives the RMS roughness of four glass beads that were sampled from the same source as the glass bead used in the experiments. As can be noted from the table, the RMS roughness of these beads ranges from 2.59 nm to 12.14 nm. Since the roughnesses of the tip and the substrate are not expected to be correlated, it is reasonable to require that the substrate’s roughness in our model be equal to the value obtained by adding together the squares of the RMS roughnesses of the tip and the substrate in the experiment and then taking the square root of that sum. As per this criteria, an appropriate range for the roughness of the substrate in our model is 3 nm to 12.2 nm, which implies that a reasonable range for *k* is (1.97, 8.03). We show the estimates for Δ*E*_*D*_ from our model for the cases when *k* = 1.97 (*σ* = 3 nm) and *k* = 8.03 (*σ* = 12.2 nm) in Fig. 8(a). While making these estimates we used *Z*_0_ as a fitting parameter. For the cases *k* = 1.97 and 8.03 we got the best fit values for *Z*_0_ to be 96 nm and 24 nm, respectively. As can be seen in Fig. 8(a), the estimates from our model for the two cases *k* = 1.97 and 8.03 are almost indistinguishable. In fact, we found that the estimates for other cases in which *k* (resp. *σ*) varied between 1.97 (resp. 3 nm) and 8.03 (resp. 12.2 nm) were also indistinguishable from the ones shown in Fig. 8(a). In each of those cases we again used *Z*_0_ as a fitting parameter. The best fit values for *Z*_0_ in these other cases varied between 96 nm and 24 nm [Fig. 8(c)]. Recall that the parameter *Z*_0_ is a measure of the distance of the inter-body cohesive forces and that we were unable to clearly identify it in the experiments of Kesari *et al*.^6^. However, the parameter *Z*_0_ has been found in other experiments to range from 10 nm to 100 nm (see Table 1). Thus, the best fit values for *Z*_0_ in our comparision lie well within the experimentally reasonable range.

**Table 1:** Estimates for the range of Z_0_ from literature.

Materials	Geometry	Range
Silica–Silica^46^	Sphere (of radius 3.8 *μ*m)–Plate	10 nm
Polystyrene–Glass^47^	Sphere (of radius 6 *μ*m)–Plate	20–100 nm

Consider the quantity

19 $${\overline{{\rm{\Delta }}E}}_{D}=\frac{{\rm{\Delta }}{E}_{D}}{{\rm{\Delta }}{A}_{c}}\approx \frac{{\rm{\Delta }}{E}_{D}}{4{R}_{t}|{h}_{{\rm{m}}{\rm{i}}{\rm{n}}}|},$$

which is the depth-dependent energy loss per unit nominal contact area. This quantity is a constant in our model, since in it Δ*E*_*D*_ depends linearly on |*h*_min_| on account of Δ*E*_*D*_ depending linearly on Δ*A*_*c*_, and Δ*A*_*c*_ depending linearly on |*h*_min_|. Figure 8(b) shows the values of ${\overline{\Delta E}}_{D}$ in the experiments at different |*h*_min_|. We computed these values using the data shown in Fig. 8(a). As can be seen, ${\overline{\Delta E}}_{D}$ is essentially a constant equal to 0.0241 J/*m*^2^ in the experiments. Thus, our model’s prediction that Δ*E*_*D*_ varies linearly with |*h*_min_| is in good agreement with experimental measurements.

## Effect of *σ* and *Z*_0_ on Δ*E*_*D*_

Figure 8(c) shows the contour plot ${\overline{{\rm{\Delta }}E}}_{D}$ of as a function of *Z*_0_ and *σ* based on our model’s prediction. It can be noted from the figure that at a fixed *Z*_0_, ${\overline{{\rm{\Delta }}E}}_{D}$ and hence Δ*E*_*D*_ decreases and approaches zero as *σ* increases. This behavior is in agreement with the trend of Δ*E* decreasing with roughness at large surface roughness regime reported by Kesari *et al*. and others, as discussed in the Introduction. Also can be seen in Fig. 8(c) is the trend that, for a fixed *σ*, ${\overline{{\rm{\Delta }}E}}_{D}$ and hence Δ*E*_*D*_ decrease as the adhesive interaction length-scale *Z*_0_ increases. We are unaware of any experimental data that can be used to check the validity of this theoretical prediction of our model. However, this trend is consistent with the numerical results reported by Song *et al*.^24^, in which the strength of adhesion decreases as the adhesive interaction length-scale increases.

## Concluding Remarks

We generated predictions from our model in the context of the experiments reported by Kesari *et al*. In general, however, it is challenging to determine *a priori* whether or not it is reasonable to apply our model to a particular contact scenario. The reason is that we assumed in our model that the contact region is multiply connected and that there is no interaction between neighboring asperities. These are reasonable assumptions only if the size of the contact region formed at each asperity is much smaller than the separation between neighboring asperities. However, we are not aware of any general criteria/models that can be used to gather information in this regard without actually solving for the complex stress and displacement fields at the contact interface. Therefore, a general theory of the type developed by Johnson^36^ that yields information about the topology of the contact region would form a valuable supplement to our model.

Kesari *et al*.^6^ and we assume that the PDMS substrates’ RMS roughness is proportional to that of their respective Si molds. Kesari *et al*. used the soft-lithography technique developed by Hua *et al*.^37^ to cast their PDMS substrates. Hua *et al*. demonstrated that their technique was capable of copying surface features as small as 3 nm from a Si mold onto a PDMS substrate. However, it has been argued that in soft-lithography it can be challenging to replicate features due to surface stress flattening out features having high curvature^38–40^. This raises the question of how justified it is to assume that the PDMS substrates’ RMS roughness is proportional to that of their respective Si molds. Some preliminary insight into addressing this question can be obtained by considering the model presented by Style *et al*.^40^ in which the surface topography of a compliant solid is taken to have a sinusoidal profile with wavelength *λ* in a single direction. As per their model, the surface stress would flatten out the sinusoidal surface if $\lambda \ll {\ell }_{{\rm{ec}}}$, where ${\ell }_{{\rm{ec}}}\,:\,=\gamma /E$ is the elasto-capillarity length, and *γ* and *E* are, respectively, the surface stress and Young’s modulus of the complaint solid. Assuming that PDMS’ Poisson’s ratio is 0.5 we get that *E* in Kesari *et al*.’s experiments is ≈0.6 MPa. Kesari *et al*. do not report the surface stress of the PDMS they use in their experiments^6^. In other experiments, however, the surface stress of PDMS was found to lie in the 15 to 50 mN/m range^41–43^. If we assume that PDMS’ surface stress in Kesari *et al*.’s experiments too lies in this range then we get that in their experiments ${\ell }_{{\rm{ec}}}$ lies in the 25–80 nm range. We found that the average distance between each asperity and its nearest neighbor on the Si mold surface shown in Fig. 7(b) is ≈17 nm (See §4 of Supplementary Material for details). Thus, at least as per the sinusoidal surface stress model, it is justified to question the validity of the assumption that a Si mold’s topography is faithfully reproduced in the PDMS substrate cast on it in Kesari *et al*.’s experiments. Thus, this preliminary analysis of the flattening effect of surface stress adds to the importance of using direct measurements of the substrate’s topography in any future comparisons of our model with experiments.

We conclude by noting that our model bears some similarities with the models presented in refs^44,45^. In particular, following Fuller and Tabor’s^23^ approach, Wei *et al*.^44^ investigated the effect of roughness on adhesion hysteresis. However, there are significant differences between their models and ours. For example, Wei *et al*. assumed the asperities’ radii of curvatures to be a constant, whereas in our model the asperities have different radii of curvatures depending on their heights. In Wei *et al*.’s model the asperity level contact is modeled using the JKR theory, whereas we model that interaction using the MD theory. Finally, Wei *et al*.’s model does not capture the depth-dependent nature of the hysteretic energy loss. Our model provides a semi-analytical formula to estimate the depth-dependent energy loss.

## Supplementary information

Supplementary Material
