# Supplementary material for: Depth-dependent hysteresis in adhesive elastic contacts at large surface roughness
Source: Sci Rep. 2019 Feb 7;9:1639. doi: 10.1038/s41598-018-38212-z (PMC6367336; doi:10.1038/s41598-018-38212-z)
Supplement: Supplementary file 1 — Supplementary Material [file 41598_2018_38212_MOESM1_ESM.pdf]

Supplementary Material: Depth-dependent hysteresis in adhesive elastic contacts at large surface roughness

Weilin Deng[1] and Haneesh Kesari[1,*]

[1]Brown University, School of Engineering, Providence, RI 02912, USA
*haneesh_kesari@brown.edu

1 RMS roughness, PSD function, and spectral moments

For a rough surface whose height is described by the topography function $z : \mathbb{R}^2 \to \mathbb{R}$, its RMS roughness can be computed as

$$\sigma = \left[\frac{1}{L_1 L_2} \int_0^{L_1} \int_0^{L_2} z(x_1, x_2)^2 \, dx_1 dx_2 \right]^{1/2}, \tag{1}$$

where L_1 and L_2 are the widths of a rectangular, nominal contact region in the $\hat{\boldsymbol{e}}_1$ and $\hat{\boldsymbol{e}}_2$ directions, respectively. The auto-correlation function corresponding to z can be defined as

$$R(x_1, x_2) = \lim_{L_1, L_2 \to \infty} \frac{1}{L_1 L_2} \int_0^{L_1} \int_0^{L_2} z(x_1', x_2') z(x_1' + x_1, x_2' + x_2) \, dx_1' dx_2'. \tag{2}$$

The rough surface's PSD function $C : \mathbb{R}^2 \to \mathbb{R}$ is the Fourier transform of R, i.e.,

$$C(q_1, q_2) = \int_{-\infty}^{\infty} \int_{-\infty}^{\infty} R(x_1, x_2) e^{-i(x_1 q_1 + x_2 q_2)} \, dx_1 dx_2, \tag{3}$$

where q_1, $q_2 \in \mathbb{R}$ are termed the wavevector components and i is the imaginary number $\sqrt{-1}$. Alternatively, the PSD function can also be computed as

$$C(q_1, q_2) = \lim_{L_1, L_2 \to \infty} \left[\frac{1}{L_1 L_2} \int_0^{L_1} \int_0^{L_2} z(x_1, x_2) e^{-i(x_1 q_1 + x_2 q_2)} \, dx_1 dx_2 \right]^2. \tag{4}$$

The PSD function's spectral moments are defined as

$$m_{k\,l} = \int_{-\infty}^{\infty} \int_{-\infty}^{\infty} C(q_1, q_2) q_1^k q_2^l \, dq_1 dq_2, \tag{5}$$

where k, $l \in \mathbb{N}$. In isotropic surfaces, the PSD function only depends on the wavenumber $q = \sqrt{q_1^2 + q_2^2}$. In this case, the isotropic PSD function C^{iso} is defined as

$$C^{\text{iso}}(q) := \frac{1}{2\pi} \int_0^{2\pi} C(q\cos\theta, q\sin\theta) \, d\theta. \tag{6}$$

Furthermore, for isotropic surfaces $m_{k\,l} = m_{l\,k}$. Therefore, Nayak [1] introduced the spectral moments $m_n := m_{n\,0} = m_{0\,n}$, $n = 0$, 2, and 4. These moments can be computed from C^{iso} as

$$\begin{aligned} m_n &= \int_0^{\infty} \int_0^{2\pi} C^{\text{iso}}(q)(q\sin\theta)^n q \, d\theta dq, \\ &= \frac{2\sqrt{\pi}\Gamma((1+n)/2)}{\Gamma(1+n/2)} \int_0^{\infty} q^{n+1} C^{\text{iso}}(q) \, dq, \end{aligned} \tag{7}$$

where Γ is the Gamma function.

In practice, the topography function z is measured only at a discrete set of points that are positioned on a rectangular grid. The points are equally spaced with intervals $\Delta x_1 = L_1/N_1$ and $\Delta x_2 = L_2/N_2$ in the $\hat{\boldsymbol{e}}_1$ and $\hat{\boldsymbol{e}}_2$ directions, respectively. The positive integers N_1 and N_2 denote the total number of points in the two directions, respectively. We denote the values of z measured at the discrete set of points as $\tilde{z}(m,n)$, $m = 0, 1, ..., N_1 - 1$ and $n = 0, 1, ..., N_2 - 1$. In concurrence with our definition of z, the datum in the $\hat{\boldsymbol{e}}_3$ direction is chosen such that these discrete set of values have a zero mean. Assuming that the surface topography is periodic with periodicities L_1 and L_2 in the $\hat{\boldsymbol{e}}_1$ and $\hat{\boldsymbol{e}}_2$ directions, respectively, we compute the discrete Fourier transform of $\tilde{z}(m,n)$ as

$$Z(k,l) = \Delta x_1 \Delta x_2 \sum_{m=0}^{N_1-1} \sum_{n=0}^{N_2-1} \tilde{z}(m,n) e^{-i2\pi(mk/N_1 + nl/N_2)}, \tag{8}$$

where $k = 0, 1, ..., N_1 - 1$ and $l = 0, 1, ..., N_2 - 1$. It can be shown using eq. (4) that

$$C(q_k, q_l) \approx \tilde{C}(q_k, q_l) := \frac{|Z(k,l)|^2}{L_1 L_2}, \tag{9}$$

where $q_k = 2\pi k/L_1$ and $q_l = 2\pi l/L_2$ are termed the discrete wavevector components, and $\tilde{C}$ is called the discrete PSD function. Similarly, using eq. (6) it can be shown that

$$C^{\text{iso}}(q) \approx \tilde{C}^{\text{iso}}(q), \tag{10}$$

where the value $\tilde{C}^{\text{iso}}(q)$ is obtained by averaging the value of $\tilde{C}$ over all pairs (q_k, q_l) for which $\sqrt{q_k^2 + q_l^2} = q$. The ordinates of the discrete set of points shown in Fig. 7 (d) of the main text are the values of the function $\tilde{C}^{\text{iso}}$. We assume that the PSD function in the experiments is the continuous, power-law PSD function given in eq. (16) of the main text. We choose the parameters defining the power law PSD function in such a way so as to make the function's values as close as possible to the values of $\tilde{C}^{\text{iso}}$.

2 Nominal contact area as per the JKR theory

For computing the nominal contact area ΔA_c in Kesari *et al.*'s experiments, we ignore the roughness of both the tip and the substrate and model the loading branch of the experiments using the JKR theory. The indentation-depth, h, and the contact radius a, in the loading phase of the experiments are then related as

$$\bar{h} = -\bar{a}^2 + (2\ell_{\text{JKR}}\bar{a})^{1/2}, \tag{11}$$

where $\bar{h} = h/R_t$, $\bar{a} = a/R_t$, and $\ell_{\text{JKR}} = \pi w/E^* R_t$. The parameters w, E^*, and R_t are defined in the Theory section of the main text. We denote the radii of the contact regions in the loading phase of the experiment when $\bar{h} = 0$ and $\bar{h}_{\min}$ as $\bar{a}_0$ and $\bar{a}_{h_{\min}}$, respectively, where $\bar{h}_{\min} := h_{\min}/R_t$. In terms of these radii the contact areas $A_c^{h_{\min}}$ and A_c^0, defined in the section of Theory of the main text, are $\pi\bar{a}_{h_{\min}}^2 R_t^2$ and $\pi\bar{a}_0^2 R_t^2$, respectively. It follows then from eq. (10) of the main text and eq. (11) that

$$\Delta A_c = \pi R_t^2 |\bar{h}_{\min}| \left[1 + \frac{(2\ell_{\text{JKR}}\bar{a}_{h_{\min}})^{1/2} - (2\ell_{\text{JKR}})^{2/3}}{\bar{a}_{h_{\min}}^2 - (2\ell_{\text{JKR}}\bar{a}_{h_{\min}})^{1/2}} \right]. \tag{12}$$

By fitting the loading branch of the the experimental P–h curves shown in Fig.2 (a) of the main text to the JKR theory we found that $w = 20$ mJ/m^2 and $E^* = 0.75$ MPa. Recall that the radius of the glass bead (tip) in the experiments is $R_t = 25$ μm. Thus, we have $\ell_{\text{JKR}} = 3.35 \times 10^{-3}$. In the experiments $|h_{\min}|$ ranges from 0 to 1500 nm. For this variation in $h_{\min}$, we found numerically from eq. (11) that $\bar{a}$ varies from 0.189 to 0.327. Consequently, the term in the square bracket in eq. (12) varies from 1.19 to 1.33, which has a variation of only $\sim 10\%$. Therefore, we approximate this term as 1.26, and from eq. (12) we obtain that

$$\Delta A_c \approx 4R_t|h_{\min}|. \tag{13}$$

3 The RMS roughness of the glass bead

In Table 1 we report the RMS roughness of four glass beads. As can be noted, the beads' RMS roughness ranges from 2.59 nm to 12.14 nm. The RMS roughness of the glass beads is 6.41 ± 4.18 nm (mean $\pm$ standard deviation). Figure 1 shows a microscope image taken during the AFM topography measurements. The AFM cantilever (white beam in Fig. 1) in those measurements has an atomically sharp, silicon nitride tip. These beads (dark spheres in Fig. 1) were sampled from the same population as from which the bead used in the experiments reported by Kesari *et al.* [2] was taken. The surface topography of the bead that is labelled as Bead #3 in Table 1 is shown in Fig. 7 (a–b) of the manuscript.

Table 1: The RMS Roughness of the glass beads.

Bead #	1	2	3	4
RMS roughness (nm)	6.68	2.59	12.14	4.21

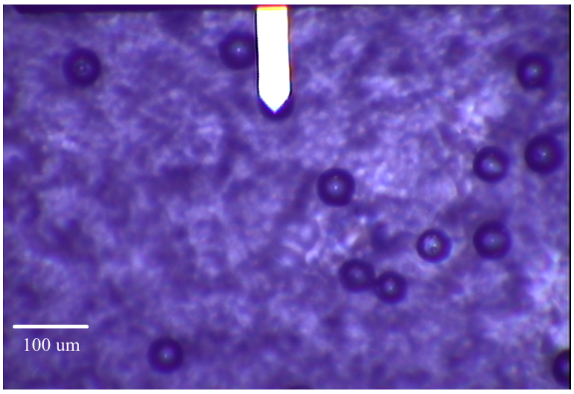

Figure 1: Silicon cantilever on the top of a 50 μm-diameter bead in double-sided Scotch Tape.

4 The average of the distance between each asperity and its nearest neighbor on the Si mold's surface

Figure 2 shows a representative region of the Si mold's surface which is shown in Fig. 7 (c) of the manuscript. We identified all the local peaks of the asperities over the Si mold's rough surface in Fig. 7 (c). The local peak is defined as the highest point around a neighbor region, as shown in red dots of Fig. 2. We calculate the average of the distance between each asperity and its nearest neighbor to be 17 nm. This value is very consistent with the correlation length ($L = 20.1$ nm) of the Si mold's rough surface that is obtained from the PSD fitting of the surface power spectrum.

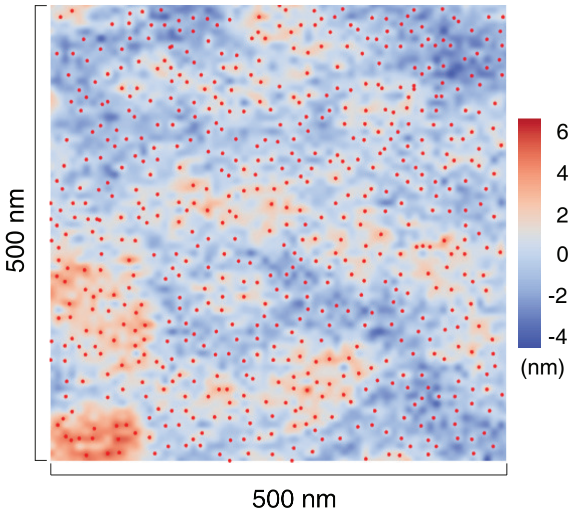

Figure 2: The local peaks (red dots) over a partial region of the Si mold's rough surface. The color denotes the height of the surface.

References

[1] P Ranganath Nayak. Random process model of rough surfaces. *Journal of Tribology*, 93(3):398–407, 1971.

[2] Haneesh Kesari, Joseph C Doll, Beth L Pruitt, Wei Cai, and Adrian J Lew. Role of surface roughness in hysteresis during adhesive elastic contact. *Philosophical Magazine Letters*, 90(12):891–902, 2010.
